# Cyclin dependent kinase 4/6 inhibitor palbociclib synergizes with BCL2 inhibitor venetoclax in experimental models of mantle cell lymphoma without *RB1* deletion

**DOI:** 10.1186/s40164-024-00499-2

**Published:** 2024-03-25

**Authors:** Diana Malarikova, Radek Jorda, Kristyna Kupcova, Jana Senavova, Alexandra Dolnikova, Eva Pokorna, Dmitry Kazantsev, Kristina Nozickova, Dana Sovilj, Celine Bellanger, David Chiron, Ladislav Andera, Vladimir Krystof, Miroslav Strnad, Karel Helman, Magdalena Klanova, Marek Trneny, Ondrej Havranek, Pavel Klener

**Affiliations:** 1 Institute of Pathological Physiology, First Faculty of Medicine, Charles University, Prague, Czech Republic; 2 First Department of Internal Medicine – Hematology, First Faculty of Medicine, General University Hospital, Charles University, Prague, Czech Republic; 3 Department of Experimental Biology, Faculty of Science, Palacky University Olomouc, Olomouc, Czech Republic; 4 BIOCEV LF1 - Biotechnology and Biomedicine Centre, First Faculty of Medicine, Charles University, Prague, Czech Republic; 5 Institute of Biotechnology, Czech Academy of Sciences, Prague, Czech Republic; 6 Integrated Research Center in Immunology and Oncology, CRCI2NA, Nantes University, Nantes, France; 7 Institute of Molecular Genetics, Czech Academy of Sciences, Prague, Czech Republic; 8 Laboratory of Growth Regulators, Palacky University Olomouc and Institute of Experimental Botany, The Czech Academy of Sciences, Olomouc, Czech Republic; 9 Faculty of Informatics and Statistics, Prague University of Economics and Business, Prague, Czech Republic

**Keywords:** Cyclin-dependent kinase (CDK) inhibitors, Palbociclib, BCL2, Venetoclax, RB1, Mantle cell lymphoma (MCL)

## Abstract

**Background:**

Mantle cell lymphoma (MCL) is a chronically relapsing malignancy with deregulated cell cycle progression. We analyzed efficacy, mode of action, and predictive markers of susceptibility to palbociclib, an approved CDK 4/6 inhibitor, and its combination with venetoclax, a BCL2 inhibitor.

**Methods:**

A panel of nine MCL cell lines were used for in vitro experiments. Four patient derived xenografts (PDX) obtained from patients with chemotherapy and ibrutinib-refractory MCL were used for in vivo proof-of-concept studies. Changes of the mitochondrial membrane potential, energy-metabolic pathways, AKT activity, and pro-apoptotic priming of MCL cells were evaluated by JC-1 staining, Seahorse XF analyser, genetically encoded fluorescent AKT reporter, and BH3 profiling, respectively. MCL clones with gene knockout or transgenic (over)expression of *CDKN2A, MYC, CDK4*, and *RB1* were used to estimate impact of these aberrations on sensitivity to palbociclib, and venetoclax.

**Results:**

Co-targeting MCL cells with palbociclib and venetoclax induced cytotoxic synergy in vitro and in vivo. Molecular mechanisms responsible for the observed synthetic lethality comprised palbociclib-mediated downregulation of anti-apoptotic MCL1, increased levels of proapoptotic BIM bound on both BCL2, and BCL-XL and increased pro-apoptotic priming of MCL cells mediated by BCL2-independent mechanisms, predominantly palbociclib-triggered metabolic and mitochondrial stress. Loss of *RB1* resulted in palbociclib resistance, while deletion of *CDKN2A* or overexpression of *CDK4*, and *MYC* genes did not change sensitivity to palbociclib.

**Conclusions:**

Our data strongly support investigation of the chemotherapy-free palbociclib and venetoclax combination as an innovative treatment strategy for post-ibrutinib MCL patients without *RB1* deletion.

**Supplementary Information:**

The online version contains supplementary material available at 10.1186/s40164-024-00499-2.

## To the editor

Mantle cell lymphoma (MCL) is a subtype of B-cell lymphomas characterized by deregulation of cell cycle progression at the G_1_-S phase transition as a consequence of cyclin D1 overexpression [1, 2]. Palbociclib, a highly selective cyclin-dependent kinase (CDK) 4/6 inhibitor demonstrated single-agent activity in patients with relapsed and/or refractory (R/R) MCL. Venetoclax, a BCL2 inhibitor also demonstrated clinical activity in MCL, but the remissions were short calling for rational drug combinations [3–6]. We analyzed efficacy, mode of action, and predictive markers of susceptibility to palbociclib, and provide sound preclinical rationale for its combination with venetoclax.

Anti-proliferative and cytotoxic effect of palbociclib was analyzed using a panel of nine MCL cell lines (Supplemental Table 1) with promising anti-proliferative effect (median IC50 13.6 nM), however the cytotoxic effect was detected only after extremely high concentrations of palbociclib with LD50 above 10µM in all cell lines.

**Fig. 1:**
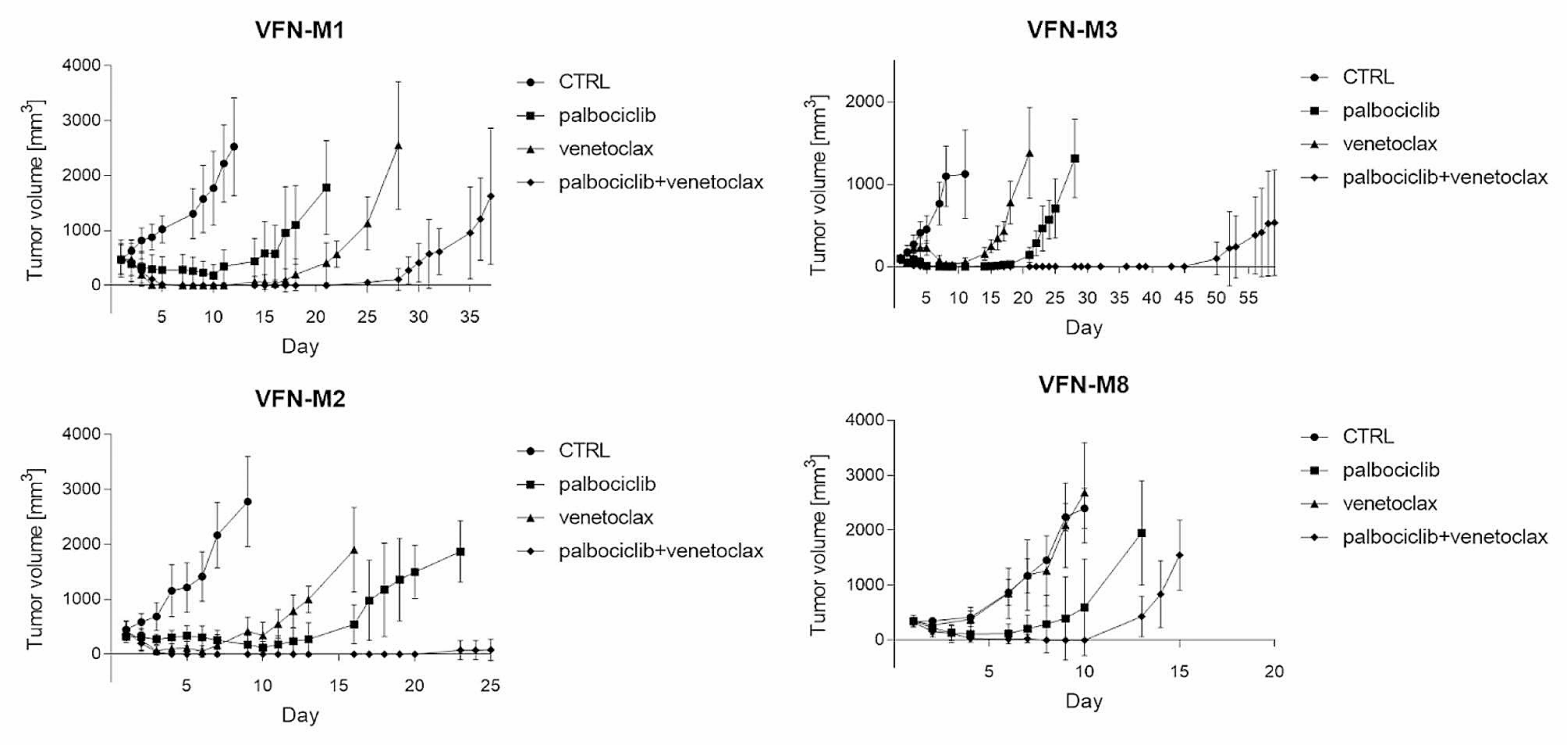
Effect of palbociclib and venetoclax on PDX models of R/R MCL. Legend: Synergistic effect of CDK4/6 inhibition with palbociclib and BCL2 blockage with venetoclax on a panel of 4 PDX models of R/R MCL. X-axis shows days from initiation of therapy. Y-axis shows calculated tumor volume of PDX tumors (means ± standard deviations)

In vitro, palbociclib increased number of cells arrested in the G1 phase, and downregulated critical cell cycle regulators including RB1 protein, while expression of CDKs and BCL2 family proteins remained without significant changes (Supplemental Figs. 1, 2 A). Despite this, in vitro screen demonstrated synergistic efficacy between palbociclib and venetoclax (Supplemental Table 2). The synergy was subsequently confirmed in vivo using a panel of 4 patient-derived xenograft (PDX) models derived from patients with R/R MCL including ibrutinib resistant cases (Fig. 1, Supplemental Table 3). Similar observations were recently reported by Yuxuan Che et al. [7]. Except for moderate downregulation of MCL1, ex vivo protein expression analysis did not significantly differ from in vitro results. Co-immunoprecipitation analyses after palbociclib revealed increased amounts of BCL2 and BCL-XL bound to BIM ex vivo and in vitro (but only after prolonged cultivation). (Supplemental Fig. 2B, 3).

Because the mode of action behind the synergy could not be explained by significant changes in the levels of free or bound BCL2 proteins, we speculated that cell cycle arrest induced by palbociclib might lead to complex changes that prime MCL cells to venetoclax-triggered apoptosis. Indeed, reactive oxygen species (ROS) and JC-1 analyses revealed increased mitochondrial ROS production and increased mitochondrial depolarization of MCL cells after exposure to palbociclib (Supplemental Fig. 4A, B). Enhanced mitochondrial proapoptotic priming by complex mechanisms independent of BCL2 proteins was confirmed by intracellular BH3 profiling (Supplemental Fig. 4C). Although the SeaHorse analysis did not reveal any specific impact of palbociclib on oxidative phosphorylation, both basal and maximal glycolytic activity was decreased in 6 out of 7 analyzed MCL cell lines (Supplemental Figs. 5 and 6). Using a genetically encoded AKT activity biosensor we demonstrated that AKT activity corresponded with the change of glycolytic activity- it was decreased in MINO and UPF1H cells but increased in JEKO-1 (Supplemental Fig. 7).

These data point to the fact that palbociclib leads to complex deregulation of key energy metabolic pathways and priming to mitochondrial apoptosis. However, precise molecular mechanisms that mediate the observed palbociclib-mediated priming of MCL cells to venetoclax remain to be elucidated.

**Fig. 2:**
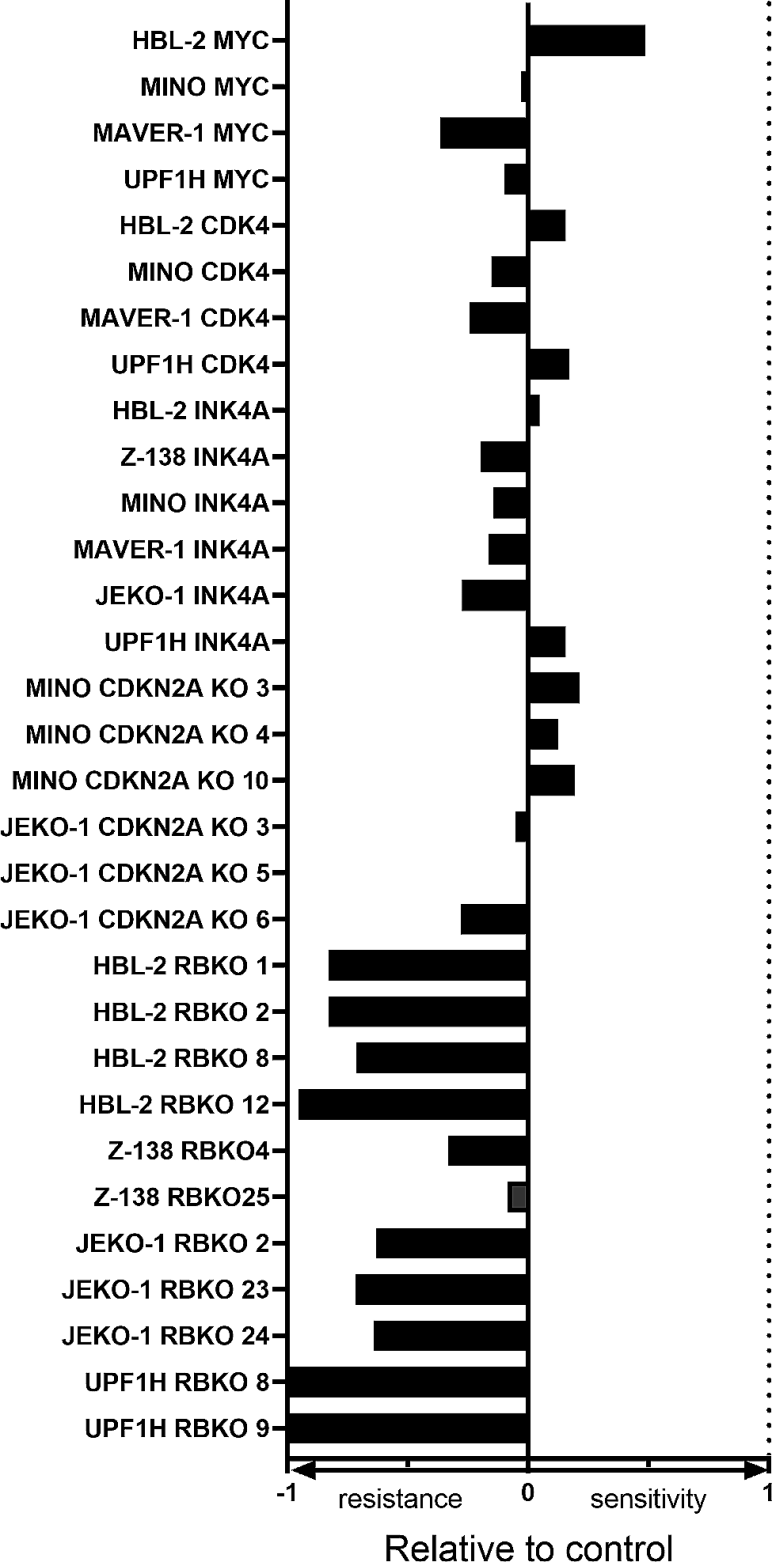
Effect of palbociclib on clones with overexpression of *MYC, CDK4, CDKN2A and K/O of CDKN2A and RB*. Legend: the figure shows relative comparison of half-maximal inhibitory concentration (IC50, in nM) in response to palbociclib between MCL clones (IC50_clone_) and corresponding controls (IC50_CTRL_). The bars were constructed according to the following formulas: 1 + IC50_CTRL_ / IC50_clone_ in the cases when IC50_clone_ > IC50_CTRL_; 1 - IC50_clone_ / IC50_CTRL_ in the cases when IC50_CTRL_ > IC50_clone_. Positive and negative bars represent more sensitive and resistant clones, respectively (compared to controls)

MCL is characterized by several other recurrent molecular / cytogenetic lesions that deregulate G1-S cell cycle transition, including deletions of *CDKN2A* or *RB1*, and amplification of *CDK4* or *MYC* genes. To evaluate the impact of these aberrations on palbociclib (and venetoclax) sensitivity, we derived MCL clones with transgenic (over)expression of p16INK4A, MYC, and CDK4, as well as MCL clones with knockout of *CDKN2A* and *RB1* genes. We demonstrated that *RB1* gene knockout leads to acquired resistance to palbociclib, which is in line with previous reports in breast cancer [8, 9]. Other tested aberrations did not significantly change sensitivity to palbociclib. Of note, overexpression of MYC was associated with increased sensitivity to venetoclax (Fig. 2, Supplemental Fig. 8).

To sum up, the innovative, chemotherapy-free combination of an FDA-approved CDK4/6 inhibitor palbociclib, and a BCL2 inhibitor venetoclax is synthetically lethal in vitro and in vivo on a panel of chemotherapy and ibrutinib-refractory MCL. Molecular mechanisms behind the observed synergy include palbociclib-triggered downregulation of anti-apoptotic MCL1, increased levels of proapoptotic BIM bound on both BCL2, and BCL-XL and increased pro-apoptotic priming mediated by BCL2-independent mechanisms, predominantly palbociclib-induced metabolic and mitochondrial stress. Deletion of *RB1* represents a predictive marker of palbociclib resistance. In translation, our data strongly support investigation of palbociclib in combination with venetoclax as an innovative treatment strategy for R/R MCL patients without *RB1* deletion.

### Electronic supplementary material

Below is the link to the electronic supplementary material.

Supplementary Material 1

## Data Availability

All data generated or analyzed during this study are included in this published article, figures and tables (and its supplementary information files). Raw data are available from the corresponding author on reasonable request.

## Abbreviations

CDK: Cyclin-dependent kinase
IC50: Half maximal inhibitory concentration
LD50: Median lethal dose
MCL: Mantle cell lymphoma
PDX: Patient derived xenograft
ROS: Reactive oxygen species
R/R: Relapsed and/or refractory

## References

[1] Wang K (2020). Cell cycle dysregulation in Mantle Cell Lymphoma: Genomics and Therapy. Hematol Oncol Clin North Am.

[2] Silkenstedt E, Linton K, Dreyling M (2021). Mantle cell lymphoma - advances in molecular biology, prognostication and treatment approaches. Br J Haematol.

[3] Davids MS (2017). Phase I first-in-human study of Venetoclax in patients with relapsed or refractory Non-hodgkin Lymphoma. J Clin Oncol.

[4] Eyre TA (2019). Efficacy of venetoclax monotherapy in patients with relapsed, refractory mantle cell lymphoma after Bruton tyrosine kinase inhibitor therapy. Haematologica.

[5] Clark AS (2016). Palbociclib (PD0332991)-a selective and potent cyclin-dependent kinase inhibitor: a review of Pharmacodynamics and Clinical Development. JAMA Oncol.

[6] Leonard JP (2012). Selective CDK4/6 inhibition with tumor responses by PD0332991 in patients with mantle cell lymphoma. Blood.

[7] Che Y (2023). Dual targeting of CDK4/6 and Bcl-2 exhibits a potent antitumor effect on mantle cell lymphoma. Blood Adv.

[8] O’Leary B (2018). The Genetic Landscape and Clonal evolution of breast Cancer Resistance to Palbociclib plus Fulvestrant in the PALOMA-3 trial. Cancer Discov.

[9] Malorni L (2016). A gene expression signature of retinoblastoma loss-of-function is a predictive biomarker of resistance to palbociclib in breast cancer cell lines and is prognostic in patients with ER positive early breast cancer. Oncotarget.

