# Supplementary material for: Cyclin dependent kinase 4/6 inhibitor palbociclib synergizes with BCL2 inhibitor venetoclax in experimental models of mantle cell lymphoma without RB1 deletion
Source: Exp Hematol Oncol. 2024 Mar 25;13:34. doi: 10.1186/s40164-024-00499-2 (PMC10962182; doi:10.1186/s40164-024-00499-2)
Supplement: Supplementary file 1 — Supplementary Material 1 [file 40164_2024_499_MOESM1_ESM.docx]

**Supplemental Data File**

**Cyclin dependent kinase 4/6 inhibitor palbociclib synergizes with BCL2 inhibitor venetoclax in experimental models of mantle cell lymphoma without RB1 deletion**

**Supplemental Figures and Tables**

|  | **IC50 [nM]** | **LD50 [µM]** |
| --- | --- | --- |
| **UPF7U** | 0.4 | 11 |
| **MINO** | 5.2 | 17.7 |
| **HBL-2** | 7.8 | 13.9 |
| **UPF1H** | 9.4 | 11.4 |
| **Z-138** | 13.6 | 11.5 |
| **GRANTA-519** | 31.5 | 15.4 |
| **JEKO-1** | 59.6 | 12 |
| **REC-1** | 211.3 | 11 |
| **MAVER-1** | 268.5 | 11 |
| **AVERAGE** | **67.5** | **12.8** |
| **MEDIAN** | **13.6** | **11.5** |

**Supplemental Table 1. Sensitivity of MCL cell lines to palbociclib.** IC50 = half-maximal inhibitory concentration, LD50 = median lethal dose

**
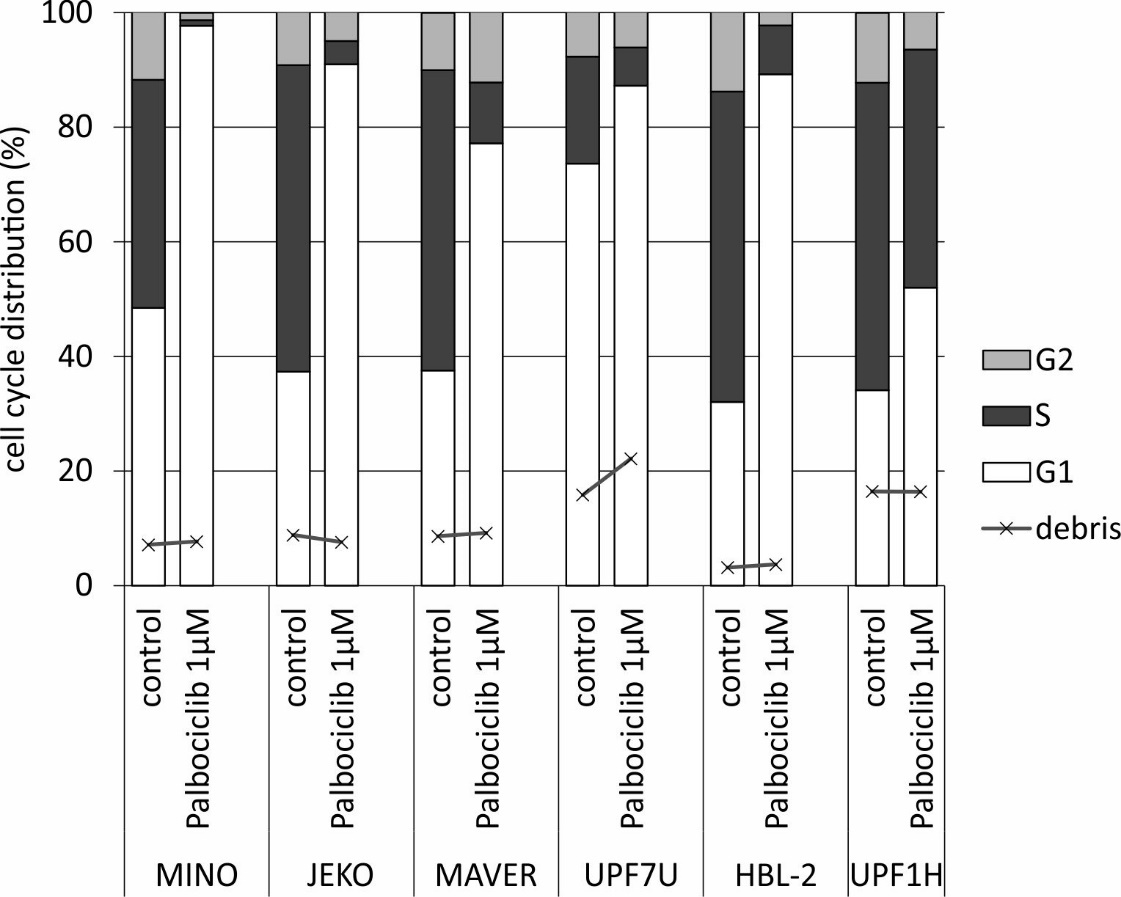
**

**Supplemental Figure 1. Increased amount of cells arrested in G1-S phase after 24-hour palbociclib exposure.** Figure shows distribution of MCL cells in different stages of cell cycle before and after 24 hour palbociclib exposure.


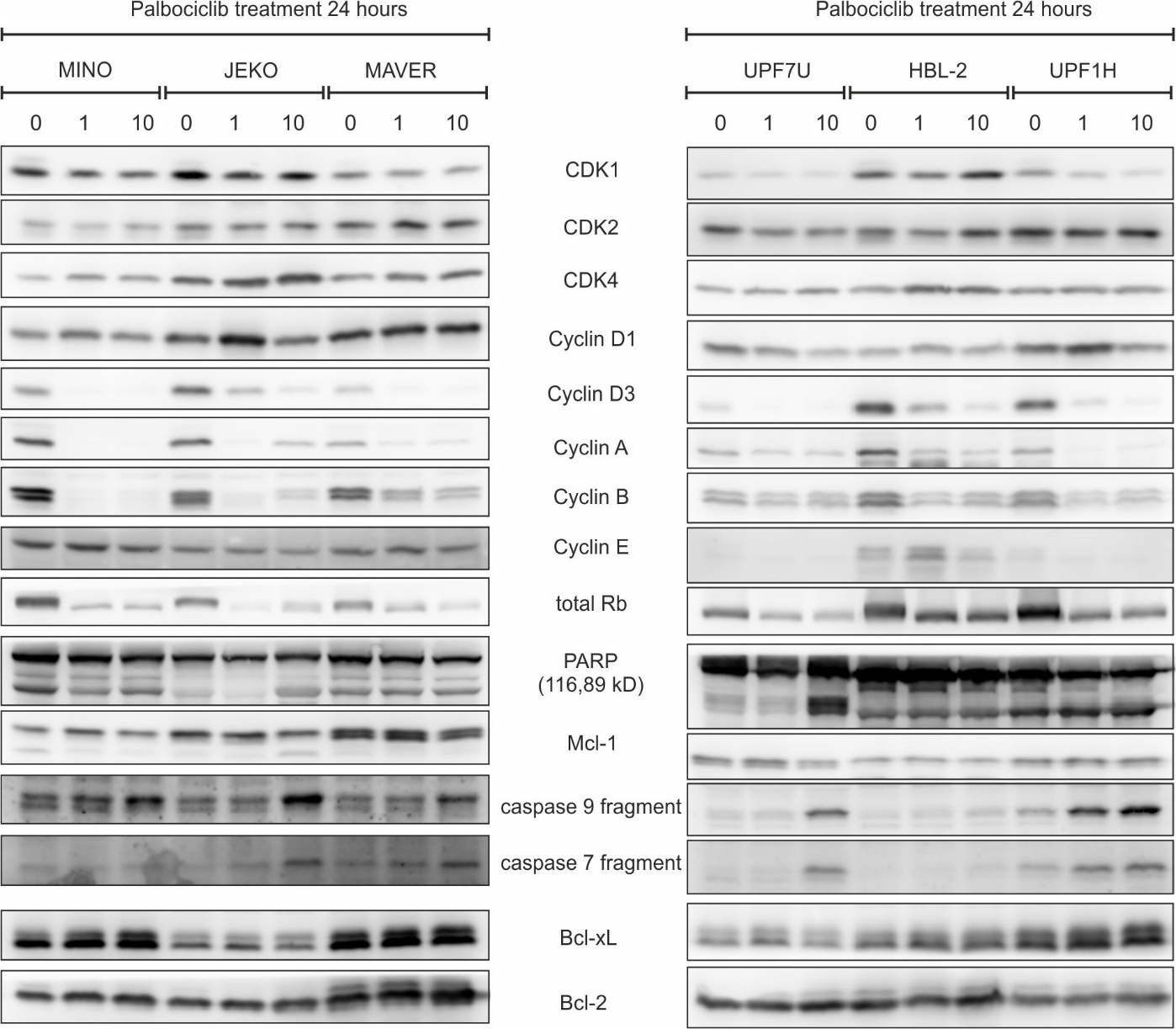
**A**

**
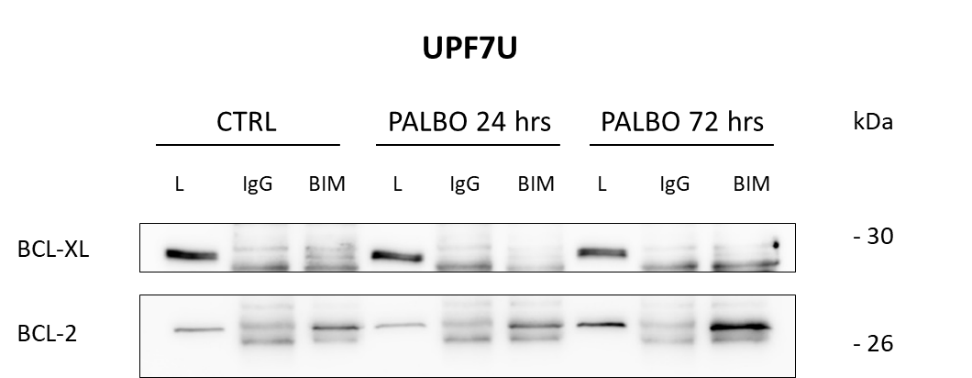
B**

**Supplemental Figure 2. Effect of palbociclib on cells *in vitro*. A Expression of regulators of cell cycle and apoptosis in MCL cell lines after 24-hour exposure to 1 µM and 10 µM of palbociclib. B Amount of BCL-XL and BCL2 bounded on BIM after 24 and 72 hours palbociclib pretreatment**

|  | **Dose Palbociclib [µM]** | **Dose Venetoclax [nM]** | **CI** |
| --- | --- | --- | --- |
| **UPF7U** | 1 | 5 | **0.13** |
|  |  | 10 | **0.11** |
|  | 10 | 5 | **0.38** |
|  |  | 10 | **0.37** |
| **MINO** | 1 | 100 | **0.80** |
|  |  | 10000 | **0.81** |
|  | 10 | 100 | **0.26** |
|  |  | 10000 | **0.15** |
| **HBL-2** | 1 | 5 | **0.99** |
|  |  | 10 | **0.85** |
|  | 10 | 5 | **0.52** |
|  |  | 10 | **0.40** |
| **UPF1H** | 1 | 10 | **0.70** |
|  |  | 100 | **0.75** |
|  | 10 | 10 | **0.79** |
|  |  | 100 | **0.32** |
| **Z-138** | 1 | 100 | **0.30** |
|  |  | 1000 | **0.17** |
|  | 10 | 100 | **0.95** |
|  |  | 1000 | **0.57** |
| **GRANTA-519** | 1 | 10 | **0.32** |
|  |  | 100 | **0.39** |
|  | 10 | 10 | **0.48** |
|  |  | 100 | **0.38** |
| **JEKO-1** | 1 | 10 | **0.17** |
|  |  | 100 | **0.16** |
|  | 10 | 10 | **0.38** |
|  |  | 100 | **0.34** |
| **REC-1** | 1 | 100 | **0.56** |
|  |  | 1000 | **0.76** |
|  | 10 | 100 | **0.15** |
|  |  | 1000 | **0.83** |
| **MAVER-1** | 1 | 5 | **0.91** |
|  |  | 10 | **0.93** |
|  | 10 | 5 | **0.48** |
|  |  | 10 | **0.35** |

**Supplemental Table 2. Effect of combination therapy with palbociclib and venetoclax on MCL cell lines.** Table shows combination indexes calculated by Chou-Talalay method from LD50 after 24 hour exposure to palbociclib and venetoclax (CI = combination index).

| **Differences** | **VFN - M1** | **VFN - M2** | **VFN – M3** | **VFN-M8** |
| --- | --- | --- | --- | --- |
| **CTRL vs. palbociclib** | 0.000001** | 0.000082** | 0.000049** | 0.000079** |
| **CTRL vs. venetoclax** | 0.000003** | 0.000003** | 0.000277** | (0.658492) |
| **palbociclib vs. palbociclib + venetoclax** | 0.000007** | 0.000004** | 0.000321** | 0.001949* |
| **venetoclax vs. palbociclib + venetoclax** | 0.000551** | 0.000059** | 0.003418* | 0.000406** |

**Supplemental Table 3.** **The p-values of partial t-tests about zero slope of mean tumour volume differences.** Statistical significance: * 10% and ** 1% simultaneous significance level, p-value in brackets refer to negative slope

**A**


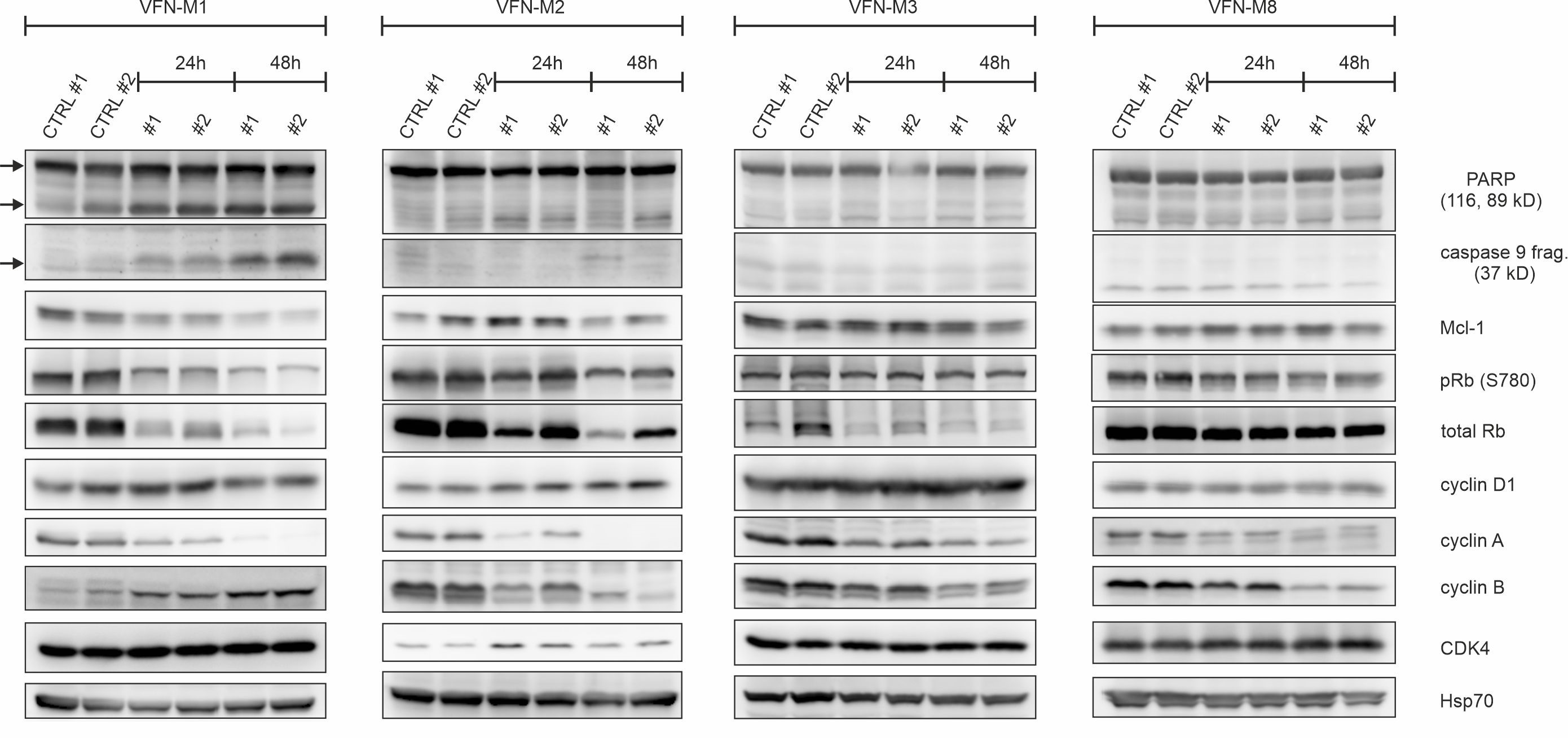


**B**

9

**
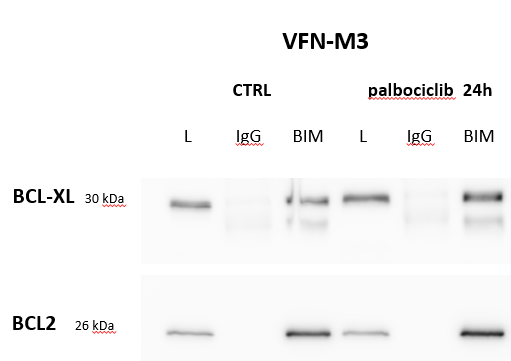
**

**C**


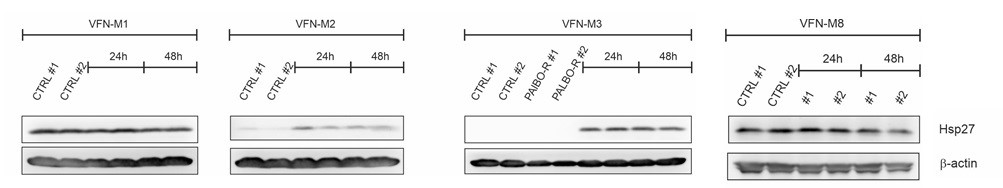


**Supplemental Figure 3. Effect of palbociclib on cells harvested from R/R MCL PDX tumors. A Expression of cell cycle and apoptosis regulators after 24 and 48 hours palbociclib pretreatment. B Amount of BCL-XL and BCL2 bound on BIM after 24 hours palbociclib pretreatment. Densitometry revealed increased amount of BIM bound on BCL-XL (1.58 times) and on BCL2 (1.27 times) after palbociclib exposure. C Levels of Hsp27 protein in VFN-M1, VFN-M2, VFN-M3 and VFN-M8 PDX tumors after 24 and 48 hours palbociclib pretreatment. Levels of Hsp27 protein in palbociclib-resistant VFN-M3 tumors (VFN-M3 PALBO-R), which were derived after prolonged exposure to palbociclib.**

9


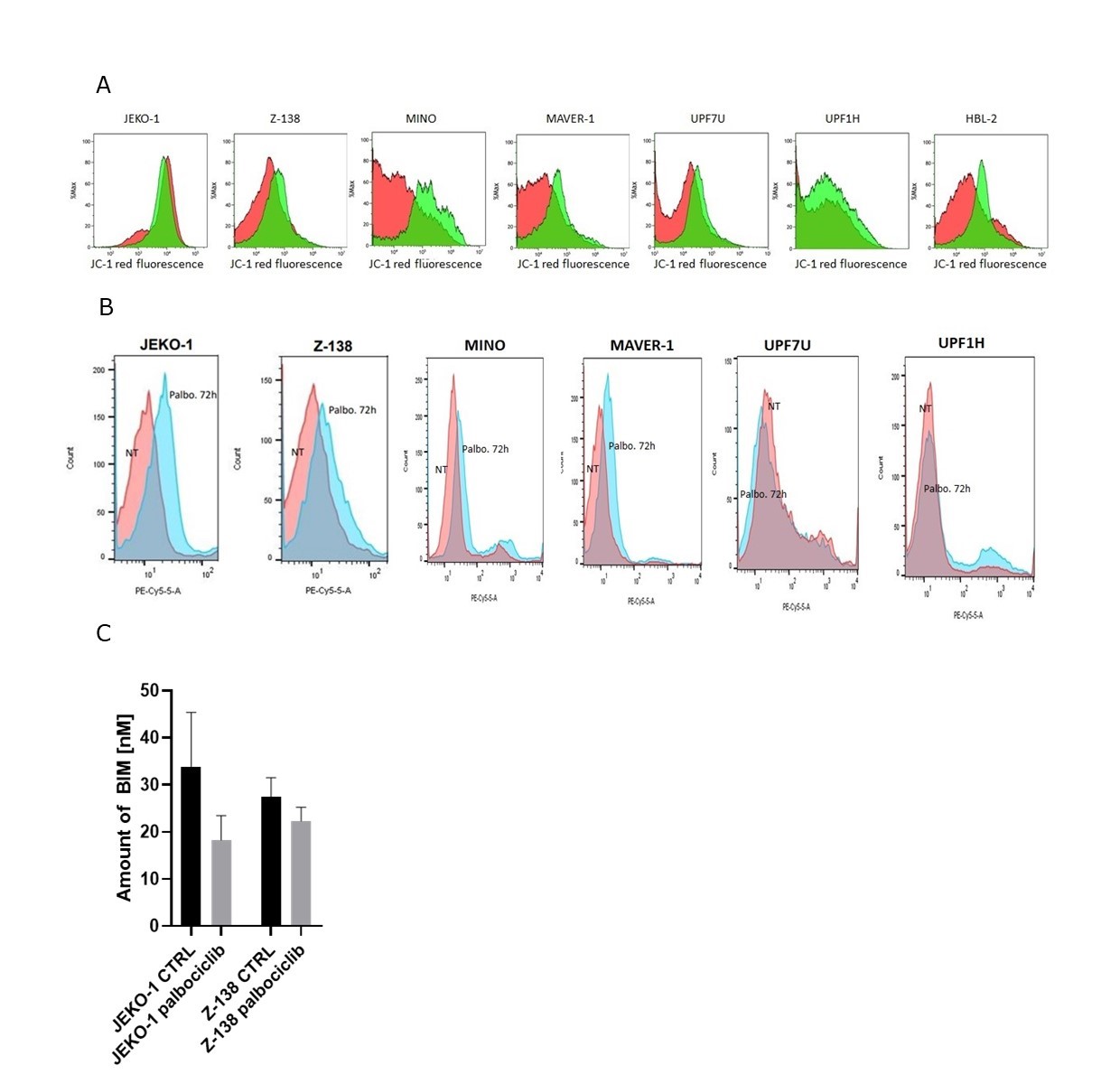


**Supplemental Figure 4. Mitochondrial changes in MCL cell lines after palbociclib exposure. A JC-1 staining after 72 hour incubation with palbociclib**. Green overlay represents cells cultivated without palbociclib. Red overlay represents cells cultivated with palbociclib. To set intensity for red fluorescence FCCP was added 20minutes before staining. **B Reactive oxygen species (ROS) levels after 72 hour incubation with palbociclib.** Red overlay represents cells cultivated without palbociclib. Blue overlay represents cells cultivated with palbociclib. **C The amount of intracellulary added BIM leading to cytochrome c release in 50% of cells after 24 hour incubation with palbociclib**

**A**

**B**

**C**

**D**

**E**

**F**

**G**

**Supplemental Figure 5. Respiratory changes after 24 and 72 hours palbociclib exposure in MCL cell lines measured by SeaHorse analyser. A Mito stress test course in time in JEKO-1 cell line** (OCR = oxygen consumption rate). **B Mito stress test course in time in Z-138 cell line** (OCR = oxygen consumption rate). **C Mito stress test course in time in MINO cell line** (OCR = oxygen consumption rate). **D Mito stress test course in time in MAVER-1 cell line** (OCR = oxygen consumption rate). **E Mito stress test course in time in UPF7U cell line** (OCR = oxygen consumption rate). **F Mito stress test course in time in UPF1H cell line** (OCR = oxygen consumption rate). **G Mito stress test course in time in HBL-2 cell line** (OCR = oxygen consumption rate).

**A**

**B**

**C**

**D**

**E**

**F**

**G**

**Supplemental Figure 6. Changes in glycolysis and glycolytic capacity after 24 and 72 hours palbociclib exposure in MCL cell lines. A** **Glycolytic stress test course in time in JEKO-1 cell line** (ECAR = extracellular acidification rate). **B** **Glycolytic stress test course in time in Z-138 cell line** (ECAR = extracellular acidification rate). **C** **Glycolytic stress test course in time in MINO cell line** (ECAR = extracellular acidification rate). **D Glycolytic stress test course in time in MAVER-1 cell line** (ECAR = extracellular acidification rate). **E Glycolytic stress test course in time in UPF7U cell line** (ECAR = extracellular acidification rate). **F Glycolytic stress test course in time in UPF1H cell line** (ECAR = extracellular acidification rate). **G Glycolytic stress test course in time in HBL-2 cell line** (ECAR = extracellular acidification rate).


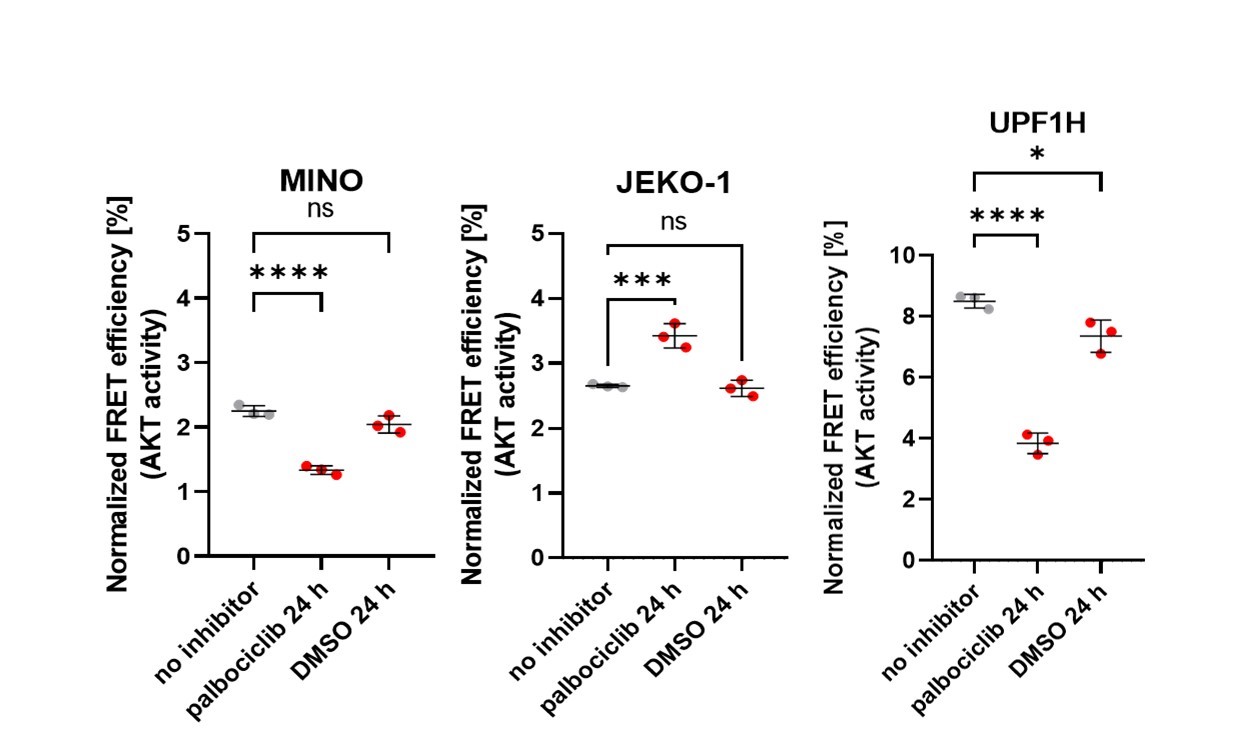


**Supplemental Figure 7. AKT activity after 24 hour incubation with 1 µM palbociclib** (FRET = Förster resonance energy transfer)

**Supplemental Figure 8. Effect of venetoclax on clones with overexpression of *MYC, CDK4, CDKN2A and K/O of CDKN2A and RB.*** The figure shows relative comparism of half-maximal inhibitory concentration (IC50, in nM) in response to venetoclax between MCL clones (IC50_clone_ ) and coresponding controls (IC50_CTRL_). The bars were constructed according to the following formulas: 1 + IC50_CTRL_ / IC50_clone_ in the cases when IC50_clone_  > IC50_CTRL_; 1 - IC50_clone_ / IC50_CTRL_ in the cases when IC50_CTRL_ > IC50_clone_. Positive and negative bars represent more sensitive and resistant clones, respectively compared to controls.

**Supplemental Methods**

**Cell lines and patient-derived xenografts**

UPF1H and UPF7U cell lines were derived in our laboratory from patients with treatment refractory MCL. The remaining cell lines were purchased from DSMZ or ATCC cell banks except for HBL2, which was a kind gift of Prof. Martin Dreyling. All commercially available cell lines were authenticated by Multiplexion. All patient-derived lymphoma xenografts (PDXs) were established in our laboratory as previously described. Genetic parameters have already been published. [1, 2] Clinical parameters are shown in Supplemental Table 4. Cell lines were cultured in Iscove's modified Dulbecco's medium (IMDM) supplemented with 15% fetal bovine serum (FBS) and 1% penicillin/streptomycin.

**Reagents, apoptosis, and proliferation assays**

Palbociclib and venetoclax were purchased from MedChemExpress. Annexin-V-FITC was from Apronex, WST8 Quick Cell Proliferation Assay was from BioVision.

Apoptosis, and proliferation were measured according to the manufacturer´s protocols as previously described [3]. Drug concentrations that induced apoptosis in 50% cells (LD50) or reduced proliferation to 50% cells (IC50) were determined by nonlinear regression algorithms using GraphPad Prism software. CompuSyn version 1.0 software (ComboSyn) was used to assess drug synergism between palbociclib and venetoclax. Combination indexes (CI) were calculated for different concentration of drugs depending on sensitivity.

**Cell cycle analysis**

Analysis of the cell cycle was carried out as previously described [4].

**Western blotting**

Western blotting was carried out as previously described [5]. Antibodies used in the study are listed in Supplemental Table 5.

**Experimental therapy of lymphoma-bearing mice**

The experimental design was approved by the Institutional Animal Care and Use Committee (MSMT-11255/2015-4 and MSMT-37330/2020-2). Immunodeficient adult female NOD.Cg-Prkdc^scid^Il2rg^tm1Wjl^/SzJ mice (referred to as NSG mice) were purchased from The Jackson Laboratory and preserved in a pathogen-free environment in individually ventilated cages, provided with sterilized food and water. Experimental therapy was implemented as previously described [1]. Venetoclax was administered by oral gavage (50 mg/kg) on days 1 - 5. Palbociclib was diluted in sodium lactate and given at 150 mg/kg by oral gavage on days 1-5 (5 hours after administration of venetoclax).

**Co-immunoprecipitation**

Cells harvested from subcutaneously grown MCL tumors 24 and 48-hours after palbociclib treatment were lysed at 4°C for 25 min in a non-denaturing lysis buffer [1% (w/v) Triton X-100, 50 mmol/L Tris-HCl (pH 7.4), 300 mmol/L NaCl, 5 mmol/L EDTA, 0.02% (w/v) sodium azide supplemented with protease inhibitor (Sigma-Aldrich)] and centrifuged (16,000 × g, 4°C, 15 min). Protein concentration of cell extracts were measured with the BCA (bicinchoninic acid) Protein Assay Kit (Pierce) according to the manufacturer’s protocol. First, protein samples were precleared with Protein A/G Agarose bead slurry (Pierce) which was incubated with anti-IgG antibody for 30 minutes at 4°C, followed by centrifugation (16,000 × g, 4°C, 2 sec). Preclearing was repeated two more times. The cell lysates were split and incubated with 10% BSA and Protein A/G Agarose beads with either a specific antibody or a corresponding isotype control immunoglobulin bound to them. Incubation took 1 hour at 4°C. Immunocomplexes were then centrifuged (16,000 × g, 4°C, 2 seconds), washed three times in ice-cold wash buffer [0.1% Triton X-100, 50 mmol/L Tris-HCl (pH 7.4), 300 mmol/L NaCl, 5 mmol/L EDTA, 0.02% sodium azide)] and once more in ice-cold 1% PBS. Subsequently, the samples were fractionated by 12 % SDS-PAGE, followed by western blotting detection, in which proteins in the gel were transferred to PVDF membrane (Bio-Rad Laboratories). To detect BCL2 and BCL-XL interaction with BIM, anti-BIM antibody (Cell Signaling) was used for the immunoprecipitation, and the precipitates were subjected to immunoblot analysis using anti-BCL2 (Dako) and anti-BCL-XL antibody (Cell Signaling).

**Oxygen consumption rate (OCR) and extracellular acidification rate (ECAR) assays**

OCR and ECAR measurements were performed using a Seahorse XFe96 analyzer (Agilent Technologies). Cells in a 6-well plate (10^6^ cfu per well) were treated with 1µM Palbociclib for 72 hours. On the day of the measurement, cells were seeded onto Corning®Cell-Tak coated XF96 cell culture microplates (5x10^4^ cells/well), in Seahorse base media and incubated at 37°C, without CO_2_ for 1 hour. Seahorse base medium of a total 180µl/well was supplemented with 500mM pyruvate, 2mM L-glutamine, 10mM glucose for OCR measurement, and with 2mM L-glutamine, for ECAR measurement. The mito stress test was performed by series of injections, starting with 1µM oligomycin (inhibits ATP-synthase), followed by 2µM CCCP (mitochondrial uncoupler, maximal respiration capacity), and a combination of 0.5µM antimycin and rotenone (inhibitors of RCIII and RCI). The glycolytic stress test was performed by series of injections starting with 10mM glucose (substrate of glycolysis), followed by 1µM oligomycin (ATP synthase inhibitor, maximal glycolysis capacity) and 50mM 2-deoxy-D-glucose (glycolysis inhibitor). OCR/ECAR were measured after each injection. After the assay, the cells were stained with Hoechst and counted using Cytation5 instrument. OCR/ECAR values are normalized based on the cell number. The effect of Palbociclib treatment on mitochondrial respiration was determined by comparing it with the respiration of non-treated cells.

**Formation of reactive oxygen species**

For generation of reactive oxygen species (ROS), 24h and 48 hours prior to the measurement 5x105 cells were seeded into a 6-well plate and treated with 1 µM palbociclib. Treatment with 250 µM H2O2 at 37°C for 4 h was used as a positive control. For ROS level evaluation, cells were incubated with MitoSox-Mitochondrial Superoxide Indicator (Thermo Fisher Scientific, Waltham, Massachusetts, United States) for 15 min at 37°C. The ROS-linked fluorescence was assessed using the PerCP-Cy5-5 (585/42) and the LSR Fortessa SORP flow cytometer (Beckton Dickenson, New York, USA). Data were analyzed using the FlowJo software.

**Mitochondrial membrane potential assessment**

Mitochondrial membrane potential was assessed by JC-1 dye (ThermoFisher) used according to the manufacturer´s protocols and measured by a flow cytometry (BD FACS Canto II) after 72 hours cultivation with or without 1µM palbociclib. JC-1 exhibits potential-dependent accumulation in mitochondria indicated by a fluorescence emission at ~529nm for the monomeric form (present at low mitochondrial membrane potential) or ~590nm for the J-aggregates (formed at higher potentials). To set gates for red fluorescence FCCP was added 20minutes before staining.

**AKT activity measurement**

AKT activity measurement using a genetically encoded FRET (Förster resonance energy transfer) – based biosensor as described in detail previously [6].

**Intracellular BH3 profiling**

Cells (previously cultivated in 15% FBS or treated with 1 µM palbociclib for 24 hours) were resuspended in DTEB buffer with 0.001% digitonin and plated at a cell density of 100,000 cells/100ul with BIM peptide (GeneCust) for 60min at 27°C. Then the cells were fixed with formaldehyde at room temperature for 15 minutes. After addition of neutralizing buffer for 5 minutes, cells were stained with anti-cytochrome C–Alexa647 (BLE612310, Ozyme) 1:40 in 2%FBS/0.1% Saponin/1%BSA/300uM NaN_3_/PBS overnight at 4°C. Loss of cytochrome c was analyzed by a flow cytometry (BD FACS Canto I).

**Establishment of MCL clones with transgenic overexpression of *MYC*, *CDK4* or *CDKN2A***

Cell lines with stably integrated cDNA carrying either *MYC* or *CDK4* were prepared using Sleeping Beauty transposon system. [7] Gene of interest was cloned into pSB vector using SfiI restriction sites. The payload also contained puromycin resistance gene and doxycycline-inducible promoter. pSB was co-transfected into MCL cells together with transposase vector SB100X (Addgene, plasmid 34879) using Neon transfection system.

Exogenous expression of INK4a was achieved using modified pLX lentiviral system. cDNA was cloned into pLX401-P2A-DsRed2 vector (derived from Addgene plasmid #121919). Resulting vectors together with vectors pMD2.G (Addgene, plasmid 12259), psPAX2 (Addgene, plasmid 12260) were introduced into HEK293T cells using Lipofectamine 3000 reagent (Thermo Fisher). After 36 h lentiviral supernatant was harvested and added to media containing MCL cells in 1:1 ratio.

In both cases cells were selected using 2 μg/ml puromycin and transgenic expression was induced by adding 0.1 μg/ml doxycycline. The overexpression was confirmed by western blotting. (Supplemental Figure 9 A - C)

**Establishment of MCL clones with *CDKN2A* or *RB1* gene deletion**

Cell lines with gene knockouts were generated according to methodology described by Cong et al [8]. Specific sgRNA targeting particular exon in a gene of interest was cloned into a pX330 recombinant plasmid (Addgene plasmid #42230) carrying Cas9 nuclease. All sgRNA sequences used in this study are listed in Supplemental Table 5. The plasmids were cloned and delivered into lymphoma cells by electroporation using Neon transfection system. For any given protein-coding exon two sgRNAs were used to create at least 70bp long deletion resulting in the knockout allele. pX330 plasmids were co-transfected with pcDNA-EmGFP plasmid. GFP-positive cells were sorted using BD FACS Aria and seeded into 96-well plate, 1 cell per well. After establishing clonal cultures gDNA and protein lysates were isolated to confirm the deletion by PCR and western blotting (Supplemental Figure 9 D, E, Supplemental Table 6).

**Statistics**

Statistical analyses were conducted in GraphPad, p value of less than 0.05 was considered as statistically significant. To assess the statistical significance of treatment effectiveness, differences between mean tumor volumes in compared groups were calculated for particular (available) time points (i.e. days) and statistical hypothesis tests of linear trend slopes equality to zero were carried out. The Bonferroni correction was used to smooth the significance level for multiple simultaneous hypothesis tests.

| **PDX model** | **Lymphoma Subtype** | **Disease Course** | **Previous Treatment** | |
| --- | --- | --- | --- | --- |
| **VFN-M1** | MCL | 1. relapse | Nordic protocol (R-maxiCHOP / HD-AraC) + ASCT | |
| **VFN-M2** | MCL | 1. relapse | R-CHOP / HD-AraC + Rituximab maintenance | |
| **VFN-M3** | MCL | 2. relapse | Nordic protocol (R-maxiCHOP / HD-AraC) + ASCT | Ibrutinib |
| **VFN-M8** | MCL | 1. relapse | R-CHOP / HD-AraC + Rituximab maintenance | |

**Supplemental Table 4. Clinical parameters of derived PDX models**

| **Target** | **Manufacturer** | **Cat. N.** | **Dilution** |
| --- | --- | --- | --- |
| INK4a | Abcam | ab108349 | 1:2000 |
| ARF | Thermo Fisher | MA5-14260 | 1:1000 |
| Rb1 | Santa Cruz Biotechnology | sc-50 | 1:1000 |
| Rabbit IgG | Jackson ImmunoRes | 711-036-152 | 1:10000 |
| Mouse IgG | Jackson ImmunoRes | 715-036-150 | 1:10000 |
| PARP-1 | Cell Signaling Technology | 9532 | 1:500 |
| Cleaved Caspase-9 (Asp330) | Cell Signaling Technology | 52873 | 1:500 |
| Caspase-7 | Cell Signaling Technology | 9492 | 1:500 |
| Mcl-1 | Cell Signaling Technology | 5453 | 1:500 |
| Bcl-2 | Merck | B3170 | 1:500 |
| Bcl-xl | Cell Signaling Technology | 2764 | 1:1000 |
| Bim | Cell Signaling Technology | 2933 | 1:1000 |
| Bak | Cell Signaling Technology | 6947 | 1:1000 |
| pRB S780 | Cell Signaling Technology | 8180 | 1:1000 |
| Rb | Cell Signaling Technology | 9309 | 1:1000 |
| cyclin A | Cell Signaling Technology | 4656 | 1:500 |
| cyclin B | Cell Signaling Technology | 4135 | 1:500 |
| cyclin D1 | Cell Signaling Technology | 2978 | 1:250 |
| cyclin D3 | Invitrogen | AHF0132 | 1:250 |
| CDK4 | Cell Signaling Technology | 2906 | 1:500 |
| c-myc | Cell Signaling Technology | 13987 | 1:1000 |
| Hsp70 | Cell Signaling Technology | 9965 | 1:1000 |
| α-tubulin | Merck | T6199 | 1:1000 |
| Akt | Cell Signaling Technology | 4691 | 1:1000 |
| pAkt (S473) | Cell Signaling Technology | 2965 | 1:1000 |
| Bax | Cell Signaling Technology | 5023 | 1:1000 |
| Bid | Cell Signaling Technology | 2002 | 1:1000 |
| mTOR | Cell Signaling Technology | 9964 | 1:1000 |
| cyclin E | Cell Signaling Technology | 4129 | 1:500 |
| CDK1 | Cell Signaling Technology | 9116 | 1:500 |
| CDK2 | Cell Signaling Technology | 2546 | 1:500 |
| CDK7 | Merck | C7089 | 1:5000 |

**Supplemental Table 5.** **List of primary and secondary antibodies**

**A**

**
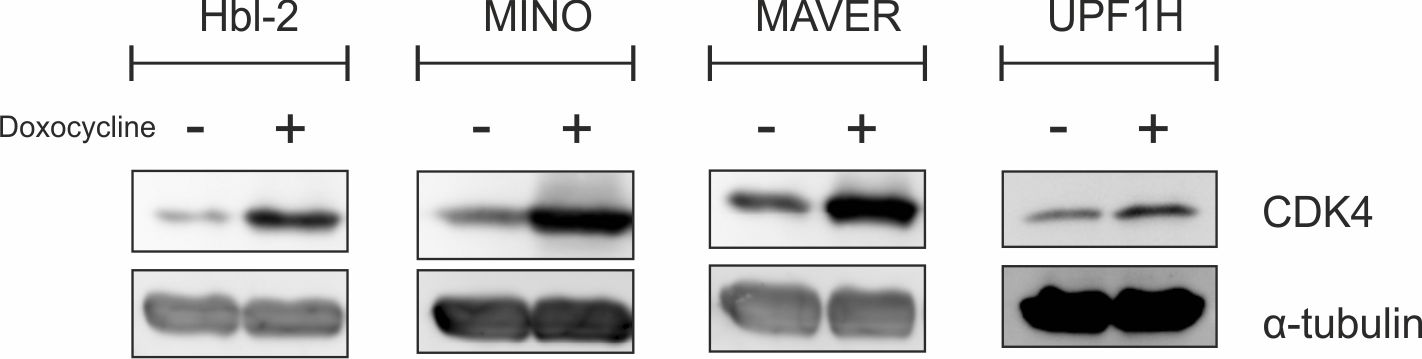
**

**B**

**
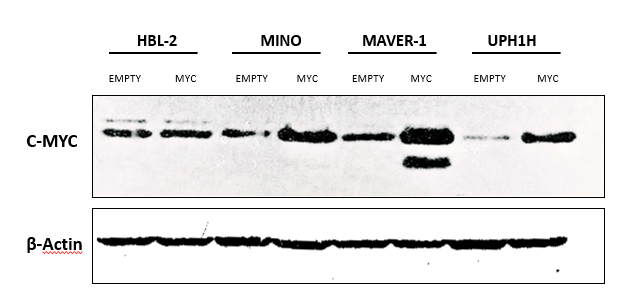
**

**C**

**
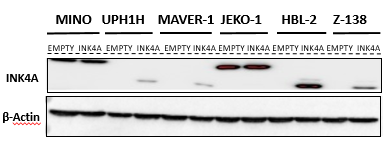
**

**D**

**
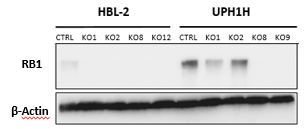

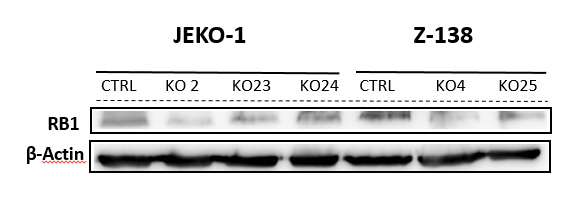
**

**E
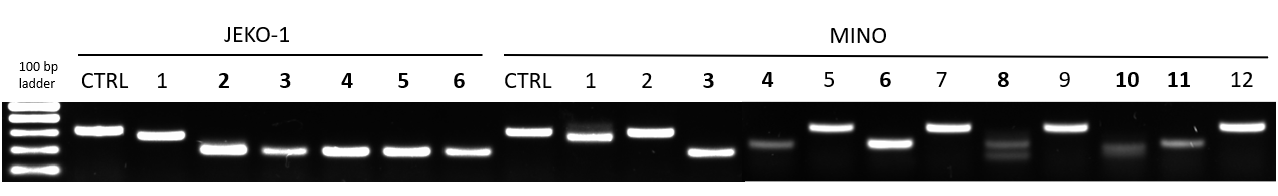
**

**Supplemental Figure 9. Validation of selected genes editing in tested cell lines. A Overexpression of *CDK4*, B Overexpression of *MYC*, C Overexpression of *INK4A*, D *RB1* K/O, E *CDKN2A* K/O.** DNA agarose gel electrophoresis showing a region containing exon 2 of *Cdkn2a* amplified using PCR. Wild-type product is 324 bp and expected K/O product is 208 bp. First lane shows 100-500 bp standard bands.

| **Gene** | **Exon** | **Cut position** | **FWD oligo** | **REV oligo** |
| --- | --- | --- | --- | --- |
| *CDKN2A* | Exon 2 | Chr9:21971014 | caccGCACGGGTCGGGTGAGAGTGG | aaacCCACTCTCACCCGACCCGTGC |
|  |  | Chr9:21971130 | caccGAGCTCCTCAGCCAGGTCCAC | aaacGTGGACCTGGCTGAGGAGCTC |
| *RB1* | Exon 3 | Chr13:48342039 | caccGTATACTATATACTACGCCAA | aaacTTGGCGTAGTATATAGTATAC |
|  |  | Chr13:48342877 | caccGCATAAATACACTTTCATAA | aaacTTATGAAAGTGTATTTATGC |

**Supplemental Table 6.** **Sequences of DNA oligos coding for single guide RNA used to produce CRISPR/Cas9 knockouts of *CDKN2A* and *RB1***. Forward and reverse oligos are hybridized resulting in a short dsDNA fragment with sticky ends for cloning into pX330 vector. Genomic position of double-stranded cuts is given using GRCh38 human genome assembly as a reference.

**References for Supplemental Methods**

1. Prukova, D., et al., *Cotargeting of BCL2 with Venetoclax and MCL1 with S63845 Is Synthetically Lethal In Vivo in Relapsed Mantle Cell Lymphoma.* Clin Cancer Res, 2019. **25**(14): p. 4455-4465.

2. Jakša, R., et al., *Complex genetic and histopathological study of 15 patient-derived xenografts of aggressive lymphomas.* Lab Invest, 2022. **102**(9): p. 957-965.

3. Klanova, M., et al., *Anti-apoptotic MCL1 Protein Represents Critical Survival Molecule for Most Burkitt Lymphomas and BCL2-negative Diffuse Large B-cell Lymphomas.* Mol Cancer Ther, 2022. **21**(1): p. 89-99.

4. Jorda, R., et al., *Selective inhibition reveals cyclin-dependent kinase 2 as another kinase that phosphorylates the androgen receptor at serine 81.* Biochim Biophys Acta Mol Cell Res, 2018. **1865**(2): p. 354-363.

5. Klanova, M., et al., *Targeting of BCL2 Family Proteins with ABT-199 and Homoharringtonine Reveals BCL2- and MCL1-Dependent Subgroups of Diffuse Large B-Cell Lymphoma.* Clin Cancer Res, 2015.

6. Henderson, J., et al., *Detecting Förster resonance energy transfer in living cells by conventional and spectral flow cytometry.* Cytometry A, 2022. **101**(10): p. 818-834.

7. Kowarz, E., D. Löscher, and R. Marschalek, *Optimized Sleeping Beauty transposons rapidly generate stable transgenic cell lines.* Biotechnol J, 2015. **10**(4): p. 647-53.

8. Cong, L., et al., *Multiplex genome engineering using CRISPR/Cas systems.* Science, 2013. **339**(6121): p. 819-23.
